# Exploring Biological Interactions: A New Pyrazoline as a Versatile Fluorescent Probe for Energy Transfer and Cell Staining Applications

**DOI:** 10.1002/open.202300092

**Published:** 2023-09-04

**Authors:** Burcu Meryem Beşer, Berat Yildirim

**Affiliations:** ^1^ Faculty of Arts and Sciences Department of Chemistry Erzincan Binali Yıldırım University Erzincan Türkiye

**Keywords:** Biophysics, cell imaging, cell staining, cytotoxicity, FRET, thermodynamic

## Abstract

Fluorescent dyes are used in biological systems, because they are highly sensitive and selective. In this work, we investigated the fluorescent probe properties of 2‐(5‐(pyridin‐2‐yl)‐1H‐pyrazol‐3‐yl) phenol (PYDP) in two media [sodium dodecyl sulfate (SDS) and human serum albumin (HSA)]. Energy transfer parameters, photophysical and thermodynamic parameters of probe were determined. We investigated cytotoxicity of PYDP against colorectal adenocarcinoma cell lines (HT‐29), breast cancer cell lines (MCF‐7) and 3T3‐L1 adipocytes (3T3 L1) cells. The cell staining property of PYDP was monitored using a confocal microscope. The results showed that PYDP binds to HSA, bindings are due to electrostatic/ionic interactions, and the binding process is spontaneous. PYDP was found to exhibit negligible cytotoxicity at high concentrations, and confocal microscope images showed that PYDP stained the cytoplasm of MCF‐7 cells.

## Graphical abstract

A novel fluorescent probe, 2‐(5‐(pyridin‐2‐yl)‐1H‐pyrazol‐3‐yl) phenol (PYDP) and its interactions with dyes in micelles as well as with HSA and its non‐cytotoxic nature have been investigated. This study verified the cell‐staining ability of PYDP for cytoplasm, opening avenues for novel fluorescent probes and cytopainters in biophysical research.

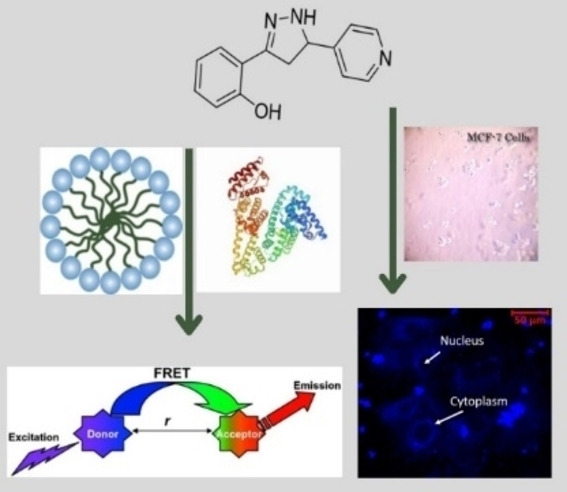

## Introduction

Fluorescent probes are mostly based on organic molecules. Fluorescence‐based techniques are widely used in biological applications due to their simple instrumentation, real‐time sensing, high sensitivity, and ease of use.^[1]^ Fluorescence microscopy is essential, especially in live‐cell imaging.^[2]^ However, in order to overcome the toxicity difficulties of the fluorescent probe to be used in this technique, the synthesis and discovery of new organic molecules that are easily synthesized, non‐toxic and have specific binding properties are important.[^3^, ^4^]

Chalcones are chromophores^[5]^ and precursors of flavonoids, which are abundant in edible plants.^[6]^ Chalcones (1) or 1,3‐diphenyl‐2E‐propene‐1‐one have an open chain structure with two aromatic rings joined by an α,β‐unsaturated carbonyl bridge.^[7]^ They can be easily obtained by Claisen‐Schmidt condensation under acidic or basic conditions.^[8]^ The synthesis of chalcone derivatives has attracted attention due to their therapeutic activities such as antitumor, anticancer, antifungal, antidiabetic activities with apparently negligible side effects.^[9]^ Chalcones are also used to synthesize heterocyclic compounds, which include pyrazolines.^[10]^ Pyrazoline derivatives exhibit blue fluorescence when excited by ultraviolet radiation.[^11^, ^12^] Therefore, pyrazoline derivatives have been extensively studied for their fluorescence properties and have been used to develop pH probes^[13]^, to detect Fe ^3+^ ions^[14]^, and hydrazine.^[15]^ In addition to their fluorescence properties, pyrazoline derivatives also have high quantum yields, making them ideal candidates for FRET studies.^[16]^

FRET is a unique energy transfer process in which energy is transferred from an excited donor fluorophore to a ground state acceptor chromophore.^[17]^ FRET is gaining popularity because it is particularly useful for measuring the molecule structure and molecular interactions, conformational changes and indications of biological events.^[18]^ The effectiveness of FRET depends on 1) the overlap of donor emission spectra and acceptor excitation spectra, 2) the distance between donor and acceptor and 3) the relative orientation of the donor emission dipole moment and the acceptor absorption dipole moment.^[19]^ The medium used in FRET is critical because the distance and orientation of the fluorophores, which are required for efficient energy transfer, are determined by the medium.^[20]^

One of the media used in FRET studies is the surfactant‐based micelle systems. They have several advantages such as high thermodynamic stability, self‐assembly, and the ability interact with both hydrophobic and hydrophilic molecules and resemble the cell membrane structure.^[21]^ Micelle systems which supposed as model membrane systems are usually utilized for the biological applications.^[22]^ Therefore, surfactant systems are often used in FRET phenomenon to study the specific interactions of fluorophores.

Human serum albumin (HSA) is a blood plasma protein that is critical for maintaining osmotic pressure in the circulation.^[23]^ Human serum albumin (HSA) is considered to be an important parameter for defining the health situation of a personal. HSA has two major and structurally important binding sites. Albumin has a hydrophobic pocket in the protein scaffold and can adjust its shape depending on the bound molecule. This property allows albumin to interact with various molecules such as proteins, glycolipids, and drugs.^[24]^ HSA is a significant vehicle in the improving of novel therapeutic agents through its abundant in the circulator system and remarkable acceptor ability.^[25]^

In this paper, we investigated the interaction of a pyrazoline derivative (PYDP) with fluorescent dyes in the micelle system and HSA in water. We also investigated at the cytotoxic effects of PYDP on both cancer cell lines and 3T3‐L1 adipocytes performed confocal microscopy imaging of breast cancer cell lines to identify the cell staining characteristics of PYDP.

## Results and Discussion

### Micelle system studies

#### Spectroscopic properties

**Figure 1:**
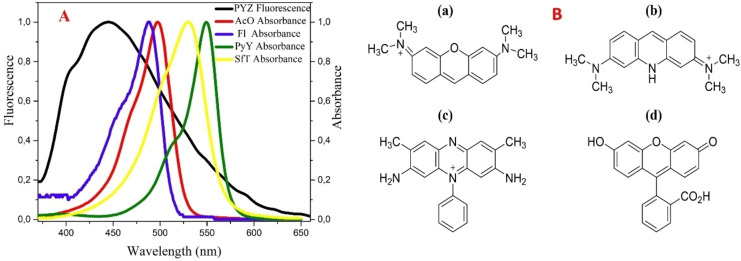
A) The emission spectrum of PYDP and the absorption spectra of AcO, Fl, PyY and SfT in SDS media (excitation wavelength 350 nm, emission wavelength range 365–690 nm).^[29]^ B) The chemical structures of fluorescent dyes [a) Pyronine Y, b) Acridine O, c) Safranine T, d) Fluorescein].

**Figure 2:**
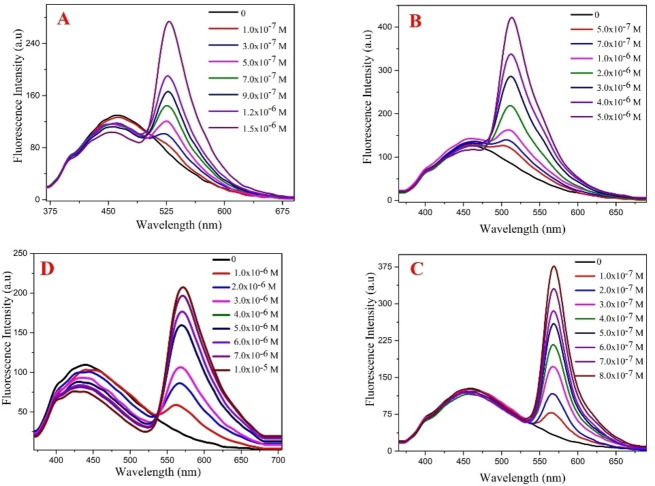
Fluorescence energy transfer spectra of PYDP in the presence of various concentrations of different acceptor dyes: (A) AcO, (B) Fl, (C) PyY and (D) SfT in SDS media.

Chalcones are bright yellow‐colored compounds found in plants and are the precursors of flavonoids.^[26]^ In this study, a chalcone‐derived synthetic pyrazoline PYDP, was evaluated for its potential as a biological fluorescent probe. In the first part of the study, we investigated the specific interactions between PYDP and four different chromophores using the FRET technique. The normalized emission spectra of PYDP excited at 350 nm and the normalized absorption spectra of AcO, Fl, PyY and SfT are shown in Figure 1. The result shows that the emission spectrum of PYDP (as a donor) and the absorption spectra of the acceptors overlap, indicating that these pairs are suitable for energy transfer studies. To confirm this, we performed FRET experiments in buffered micelle systems by increasing acceptor concentrations (Figure 2). In micelle systems, the donor molecule is distributed in the micelle scaffold and the acceptor concentration determines the probability of finding acceptor molecules in the vicinity of the donors. Increasing the concentration of acceptors increases FRET because simply there are more acceptors within the range of energy transfer.^[27]^ We observed that the intensity of the absorption band of the acceptor increases with increasing concentration of the acceptor molecule (AcO), indicating a specific interaction with high affinity between donor and acceptor (Figure 2). If the value of light absorption remains constant when the concentration of a solution is changed, that wavelength is known as the isosbestic point.^[28]^ The isosbestic points were observed at 534 nm and 536 nm for PyY and SfT, respectively, however, no isosbestic points were obtained for AcO and Fl.

#### Fluorescence quantum yields

The calculation of fluorescence quantum yield is important, because it is used to calculate quenching‐rate constants, energy transfer, and radiative and nonradiative rate constants.^[30]^ The fluorescence quantum yield (Φ) is a key property of a fluorophore and indicates the ability of the fluorophore to convert absorbed photons into emitted photons. It is expressed by the following Equation 1:

(1) $$\Phi {}_{f}=\mathrm{Number}\;\mathrm{of}\;\mathrm{emitted}\;\mathrm{photons}/\mathrm{Number}\;\mathrm{of}\;\mathrm{absorbed}\;\mathrm{photons}$$

Fluorescence quantum yield data are used to determine the efficiency of the fluorescence process.^[30]^ Two methods can be used to measure the quantum yield: the absolute method and the relative method.^[19]^ The absolute method is applicable in both solid and dissolved phases and is based on a single data collection containing the number of photons emitted by the sample. This method is useful when there is no reliable reference compound that has similar fluorescence characteristics to the compound to be determined. In addition, it requires special equipment. The relative comparison method is the most widely used method and requires a standard reference compound with a known quantum yield value. This method is relatively more accurate since it involves the solution gradient.^[19]^ To calculate the quantum yield of a compound, the integrated fluorescence intensity of the reference compound solution is plotted against the absorbance of the reference compound solution, and the sample with the unknown quantum yield is estimated from this plot. The Parker‐Rees method given in Equation (2) was used to calculate the quantum yields.^[31]^

(2) $$\varphi_{s}=\varphi_{r}\left(\frac{D_{s}}{D_{r}}\right){\left(\frac{n_{s}}{n_{r}}\right)}^{2}\left(\frac{1-10^{{-OD}_{r}}}{1-10^{{-OD}_{s}}}\right)$$

where, φ_s_ and φ_r_ s are the fluorescence quantum yields of the sample and the reference, D_s_ and D_r_ are the areas under the corrected fluorescence spectra of the sample and reference, n_s_ and n_r_ are the refractive index of the sample and reference solvent, OD_s_ and OD_r_ are the optical density measured at the excitation wavelength of the sample and reference, respectively.

A high fluorescence quantum yield is advantageous, because it allows detection of a fluorescence signal even at low concentrations.^[32]^ The fluorescence quantum yields of PYDP with Fl, PyY, AcO and SfT dyes at increasing concentrations in the micellar system (SDS) are listed in Table 1. Quinine sulfate was used as the reference compound in the quantum yield calculation (Φ=0.58).^[33]^ The fluorescence quantum yield of PYDP was 0.127 (Table 1), which is relatively low as compared to other pyrazoline derivatives reported in the literature. Previously, the quantum yielded of three novel pyrazoline compounds in chloroform were reported to be 0.83, 0.76, and 0.78.^[34]^ Moreover, quantum yields ranging from 0.09 (in water) to 0. 66 (in tetrahydrofuran) were found for the same pyrazoline compound in different solvents. Therefore, the quantum yield of PYDP could change depending on the solvent.

**Table 1:** Fluorescence quantum yield of PYDP with increasing concentration of acceptor dyes in aqueous SDS solution.

[SfT]/M	Φ_f_	[AcO]/M	Φ_f_	[PyY]/M	Φ_f_	[Fl]/M	Φ_f_
0	0.127	0	0.127	0	0.127	0	0.127
1.0×10^−6^	0.118	1.0×10^−7^	0.121	1.0×10^−7^	0.121	5.0×10^−7^	0.095
2.0×10^−6^	0.108	3.0×10^−7^	0.113	2.0×10^−7^	0.114	7.0×10^−7^	0.093
3.0×10^−6^	0.106	5.0×10^−7^	0.111	3.0×10^−7^	0.113	1.0×10^−6^	0.090
4.0×10^−6^	0.099	7.0×10^−7^	0.103	4.0×10^−7^	0.100	2.0×10^−6^	0.058
5.0×10^−6^	0.093	9.0×10^−7^	0.091	5.0×10^−7^	0.108	3.0×10^−6^	0.053
6.0×10^−6^	0.085	1.2×10^−6^	0.095	6.0×10^−7^	0.106	4.0×10^−6^	0.048
7.0×10^−6^	0.079	1.5×10^−6^	0.081	7.0×10^−7^	0.105	5.0×10^−6^	0.048
8.0×10^−6^	0.076			8.0×10^−7^	0.106		

In the fluorescence energy transfer process, the donor‘s fluorescence quantum yield decreases as the donor transfers its energy to the acceptor. Here, PYDP is the donor molecule. Moreover, the results showed that the quantum yield of PYDP gradually decreased with upon increasing concentration of acceptor dyes (Table 1), which could be attributed to the effective energy transfer from PYDP to the dyes.

#### Energy transfer parameters (J and R)

For the fluorescence energy transfer process to occur, the emission spectrum of a fluorophore, called the donor, must overlap with the absorption spectrum of another molecule, called the acceptor. The acceptor does not need to be fluorescent. The donor and acceptor are coupled by a dipole‐dipole interaction. The extent of energy transfer is determined by the distance between the donor and acceptor, and the extent of spectral overlap. For convenience the spectral overlap is described in terms of the Förster distance (R_0_). The distance at which FRET is 50 % efficient is called the Förster distance. To quantify the energy transfer efficiency of PYDP as a donor, the spectral overlap integral of the donor‐acceptor pair (J) and Förster distance (R_0_) parameters were calculated. R_0_ is given by Equation 3:

(3) $$R_{0}^{6}=8.8x10^{-25}k^{2}N^{-4}\varphi J$$

Where *κ* ^*2*^ is the spatial orientation factor of the dipole, *N* is the refractive index of the medium, *Φ* is the fluorescence quantum yield of the donor, and *J* is the overlap integral of the fluorescence emission spectrum of the donor and absorption.^[35]^ The parameter *J* represents the spectral overlap area of donor emission and acceptor absorption, which can be calculated by the Equation 4:

(4) $$J=\frac{\sum F\left(\lambda \right)\epsilon \left(\lambda \right){\sigma \lambda }^{4}\Delta \lambda }{\sum F\left(\lambda \right)\Delta \lambda }$$

Where *F(λ)* is the corrected fluorescence intensity at wavelength λ, ϵ(λ) is the molar absorption coefficient of the acceptor at wavelength λ.

The parameters of energy transfer in the micelle system were calculated and the results are shown in Table 2. The overlap of the integrals *J* for the PYDP and dye(s) pair in SDS ranged from 9.03×10^−14^ to 1.57×10^−15^. The Förster distance is usually between 2–6 nm.^[36]^ In this study, ranged from 36.85 Å and 40.22 Å for FRET pairs, indicating that energy transfer from PYDP to the dyes is highly likely to occur. FRET efficiency (E) is the fraction of photon energy absorbed by the donor and transmitted to the acceptor.^[37]^ E is based on the distance between the donor and acceptor distance (r) and was calculated using the Equation 5:

(5) $$E=1-\frac{F}{F_{0}}=\frac{R_{0}^{6}}{R_{0}^{6}+r^{6}}$$

**Table 2:** Energy transfer parameters for PYDP‐Dye(s) system.

	J [M^−1^ cm^−1^ nm^4^]	R_0_ [Å]
AcO	1.43×10^−15^	39.56
SfT	1.57×10^−15^	40.22
PyY	9.03×10^−14^	36.85
Fl	1.54×10^−15^	40.09

As can be understood, E is increased by shortening the donor‐acceptor distance (r). Table 3 shows r and the corresponding E values at different temperatures for all FRET pairs. From this, it can be seen that the PYDP‐SfT pair has the shortest distance and the highest E.

**Table 3:** Energy transfer parameters, quenching parameters and binding constant of PYDP in SDS environment.

Sample	Temperature (K)	*K*_sv_ [M^−1^]	k_q_ [M^−1^ s^−1^]	*K*_a_ [M^−1^]	E	R [Å]	n
AcO	298	5.57×10^4^	1.44×10^4^	6.42	0.330	44.52	0.153
	308	8.85×10^4^	2.28×10^4^	5.69	0.366	43.35	0.148
	318	1.59×10^5^	4.10×10^4^	1.41	0.332	44.45	0.108
Fl	298	4.54×10^4^	2.02×10^4^	11.12	0.203	50.35	0.526
	308	2.55×10^5^	1.14×10^5^	13.67	0.297	46.28	0.654
	318	2.17×10^5^	9.68×10^5^	8.57	0.337	44.88	0.372
PyY	298	6.54×10^5^	1.77×10^5^	3.61×10^2^	0.313	42.01	0.440
	308	3.81×10^5^	1.03×10^5^	2.83×10^2^	0.215	74.02	0.428
	318	7.50×10^5^	2.03×10^5^	5.02×10^6^	0.104	52.76	1.102
SfT	298	5.41×10^4^	1.40×10^4^	2.64×10^4^	0.489	40.52	0.918
	308	4.61×10^4^	1.19×10^4^	3.23×10^4^	0.192	51.10	0.958
	318	2.60×10^4^	6.72×10^3^	3.58×10^2^	0.178	51.90	0.600

#### Fluorescence quenching mechanism (K_sv_, k_q_, K_a_, E, r(Å), n)

The intensity of fluorescence can be decreased by a wide variety of processes. Such decreases in intensity are called quenching. Quenching can occur by different mechanisms. There are two quenching mechanisms, dynamic and static quenching.^[35]^ Dynamic quenching means that the complex between donor and acceptor occurs during the lifetime of the excited state, whereas static quenching occurs between quenchers and fluorophores in the ground state, and it forms forming a new compound in the ground state.^[38]^ In the static quenching mechanism, the stability of the formed compound decreases with increasing temperature, because the high temperature disassociates weakly formed intermolecular bonds.^[39]^ We performed a Stern‐Volmer analysis to investigate the fluorescence quenching mechanism using the Equation 6:

(6) $$\frac{I_{0}}{I}=\frac{F_{0}}{F}=1+k_{q}\tau \left[Q\right]=1+K_{SV}\left[Q\right]$$

Where *F_0_* and *F* are the emission intensities of the quencher in the absence and presence of the quencher, respectively. *K_sv_* is the Stern‐Volmer quenching constant, which indicates the efficiency of quenching. k_q_ is the bimolecular quenching constant, *τ_0_* is the average lifetime of the fluorophore in absence of quencher and [*Q*] is the concentration of the quencher.^[40]^ To confirm the quenching mechanism, the double logarithm given by Equation (7) was used:

(7) $$log\frac{\left(F^{\circ }-F\right)}{F}=logK_{a}+n\;log\left[Q\right]$$

where (*n*) and (*K*) refer to the number of binding sites and the binding constant, respectively.

**Figure 3:**
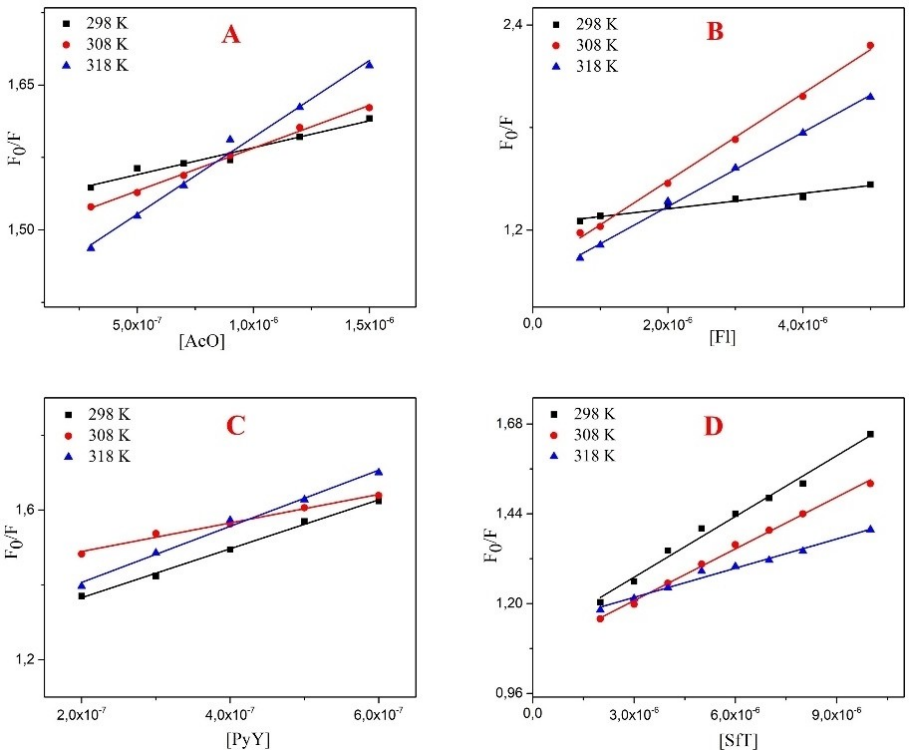
Stern‐Volmer plot of PYDP binding with (A) AcO, (B) Fl, (C) PyY and (D) SfT in SDS media.

**Figure 4:**
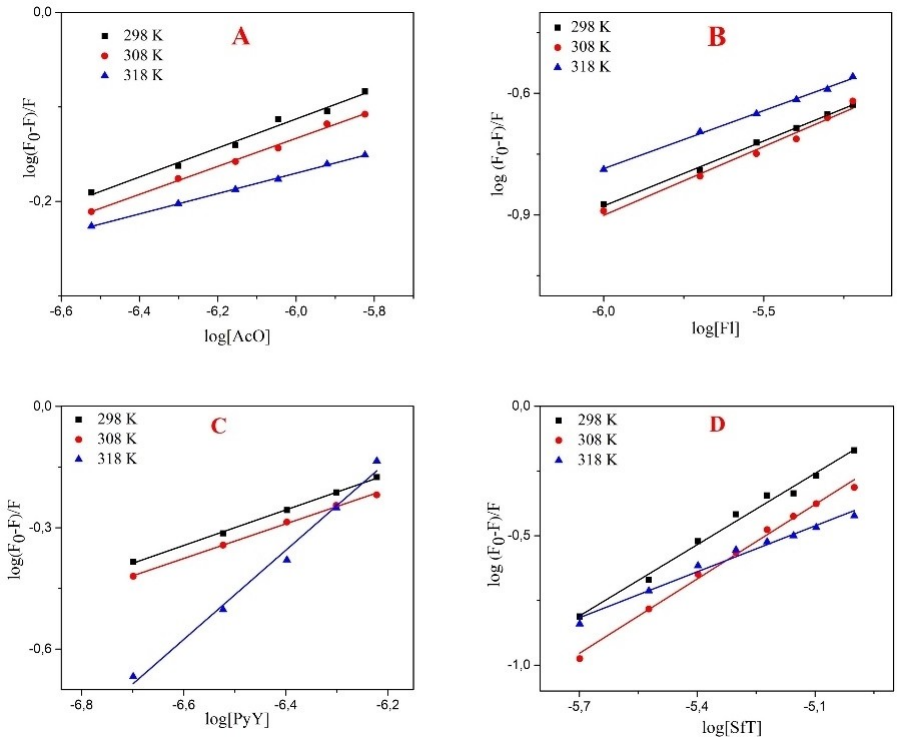
Plots of the PYDP quenching effect on (A) AcO, (B) Fl, (C) PyY and (D) SfT in SDS media at different temperatures (298, 308, and 318 K, respectively).

The plot of F°−F/F versus [Q] at different temperatures are shown in Figure 3. A plot of log(F°−F)/F vs. log[Q] forms a straight line where n is the slope and log*K* is the intercept (Figure 4). Corresponding results (*K*_*s*v_, *K*_q_, *K*_a_, E and r (A)) are listed in Table 3.

To recognize between static and dynamic quenching, their different dependence on temperature is examined. Another distinguishing parameter is fluorescence lifetime measurements. Higher temperatures cause faster diffusion and thus larger amounts of collisional quenching. Also, increasing the temperature will lead to dissociation of weakly bonded complexes and therefore less static quenching. Looking at Equation (3) and Table 3, generally the *K*_sv_ values have been rise with increasing temperature. This indicates dynamic quenching for PYDP‐dye(s) donor‐acceptor systems. As a matter of fact, FRET is by definition a dynamic quenching mechanism because energy transfer takes place from an excited donor.

The bimolecular quenching constant (k_q_) reflects the efficiency of quenching or the accessibility of the fluorophores to the quencher. Diffusion‐controlled quenching typically results in values of k_q_ near 1.0×10^10^ M^−1^ s^−1^. Values of k_q_ smaller than the diffusion‐controlled value can result from steric shielding of the fluorophore or a low quenching efficiency. k_q_ is calculated from the equation (*K*_sv_=k_q_.τ_0_) and k_q_ values were below the maximum quenching rate constant (1.0×10^10^ M^−1^ s^−1^). The values of n are not consistent with varying temperatures, which could mean that the complex between donor and acceptor is not very stable, and the effect of temperature is minor. The number of binding sites is about 0.5 for PYDP+Fl at 25 °C, which means that one mole of PYDP binds to 0.5 mol of Fl.^[40]^ The efficiency of FRET is based on the distance (r) between donor and acceptor, which should be around 10–100 Å, as previously reported.^[41]^ In this study, the calculated R values range from 40.52 Å to 74.02 Å at different temperatures for all FRET pairs, which is well within the range (Table 3).^[42]^

#### Fluorescence lifetime measurement studies

**Figure 5:**
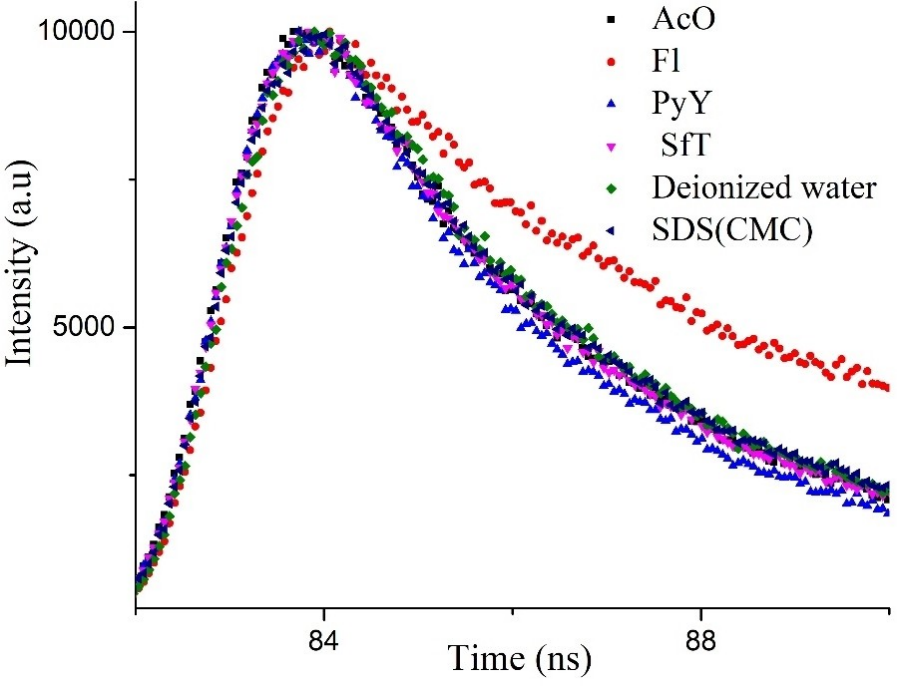
Lifetime decay profiles of PYDP determined at an excitation wavelength of 310 nm and an emission wavelength range of 460–475 nm in aqueous buffered solution and micelle.

**Figure 6:**
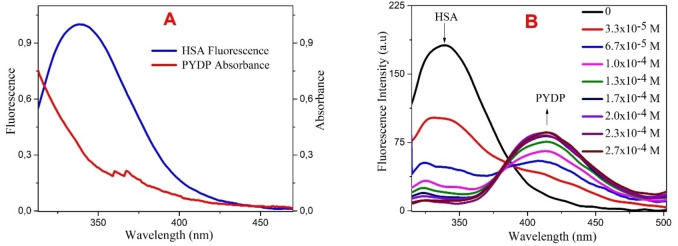
Spectra of HSA and PYDP (A) Fluorescence and absorbance spectra of HSA and PYDP in aqueous media [HSA]: 1.0×10^−6^ M, [PYDP]: 1.0×10^−4^ M. (B) Fluorescence spectral changes of HSA in response to increasing concentrations of PYDP. λ_ex_: 290 λ_em_; 305–570 nm.

The lifetime (τ) of a fluorophore is the average time between its excitation and return to the ground state. Fluorescence lifetime measurements are used to distinguish between static and dynamic quenching processes. Time‐correlated single‐photon‐counting (TCSPC) technique was used to determine the lifetime decay of PYDP. The emission rates of fluorescence are typically 10^8^ s^−1^, so that a typical fluorescence lifetime is near 10 ns (10×10^−9^ s). The nanosecond decay profiles of PYDP with different dyes at 310 excitation nm are shown in Figure 6 and the calculated values are given in Table 4. Wherein τ_1_ and τ_2_ are the lifetimes, B_1_ and B_2_ are the fluorescence lifetimes, <τ> is the fluorescence lifetime, χ^2^ is the accuracy of the measurement. The fluorescence lifetime values provide information about the interactions of the fluorescence probe with the environment. It should be noted that PYDP has given multiple lifetimes for each system (Figure 5). If there is no acceptor in close proximity to the donor, the donor is partially quenched by the acceptor and a multiexponential decay occurs. Besides, PYDP has shown a longer lifetime in SDS as than in water. This means that there is an interaction between PYDP and negatively charged SDS micelles. Time‐resolved donor decays provide a great deal of information about the purity of the sample and the distance between donor and acceptor. Moreover, the fluorescence lifetimes of the PYDP alone (in deionized water) and PYDP with dyes (in SDS) were similar to those of PYDP in SDS solution. In addition, the first lifetimes of the pairs were overall shorter than the second lifetimes, except for the pair PYDP and Fl. This may indicate a complex formation between SDS and Fl, which causes a change in the microenvironment of PYDP. Previously, it was reported that the fluorescence spectra of Fl increased with increasing SDS concentration, suggesting the formation of a complex between Fl and SDS due to hydrophobic interactions.^[43]^ The average fluorescence lifetimes (<τ>) are calculated by taking the mean values of two fluorescence lifetimes by multiplying their percentage values. There are no significant changes in the average lifetime values among the pairs. The value of χ^2^ is between 1 and 2, as expected, and the accuracy increases as the value approaches 1.

**Table 4:** Fluorescence lifetime values of PYDP in different environments.

Samples	$\tau_{1}$ [ns]	$B_{1}$ [%]	$\tau_{2}$ [ns]	$B_{2}$ [%]	<τ> [ns]	$\chi^{2}$
PYDP in water	1.706	18.31	4.407	81.69	3.912	1.569
PYDP in SDS	3.184	71.42	8.017	28.58	4.608	1.844
PYDP+AcO	1.508	18.71	4.425	81.29	3.879	1.495
PYDP+Fl	3.931	36.81	1.261	63.19	2.243	1.583
PYDP+PyY	1.568	25.53	4.417	74.47	3.689	1.495
PYDP+SfT	1.606	20.34	4.455	79.66	3.866	1.440

#### Thermodynamic parameters and the nature of the binding forces

Next, we calculated the thermodynamic parameters responsible for the binding PYDP with four different dyes using Van't Hoff equation given in Equations (8) and (9) below.^[44]^

(8) lnk=-ΔH/RT+ΔS/R

(9) ΔG=ΔH-TΔS=-RTlnk

Where R is the universal gas constant, T is the temperature in Kelvin, and K is the binding constant at the corresponding temperature. Plotting ln*k* against the 1/T plot, the values of ΔH and ΔS are calculated from the slope and cut‐off point, respectively. Based on the values of ΔH and ΔS, the nature of interaction could be determined (Table 5).

**Table 5:** Thermodynamical parameters of molecular interactions.

**Negative**	**Positive**	**Nature of biding forces**
**ΔH and ΔS**		Van der Waals and hydrogen bonds
**ΔH**	ΔS	Electrostatic interactions
	ΔH and ΔS	Hydrophobic forces
**ΔG**		Favorable (spontaneous)
	ΔG	Unfavorable

The results showed that the interaction between PYDP and other fluorescent dyes was stable and spontaneously driven by negative ΔG^0^ values (Table 6). All donor‐acceptor pairs exhibited negative ΔH and positive ΔS values, suggesting that all interactions were due to the electrostatic and ionic interactions.

**Table 6:** Thermodynamic parameter values for the interaction of PYDP with different fluorescence dyes.

	ΔH^0^ [kJ mol^−1^]	ΔS^0^ [kJ mol^−1^ K^−1^]	ΔG^0^ [kj mol^−1^]
AcO	−24.54	0.08	−47.50
Fl	−166.84	0.58	−325.22
PyY	−367.19	1.26	−711.11
SfT	−128.15	0.46	−253.89

### HSA studies

So far, we have discussed the suitability of PYDP as a fluorescent donor probe for biological systems. In this section, we have studied the interaction between HSA (donor) and PYDP (acceptor) in the aqueous environment. HSA have been used as a model protein for many and diverse biophysical and physicochemical studies. The intrinsic fluorescence feature of HSA is due to two types of amino acids: one is tryptophan in subdomain IIA (Trp‐214) and the other is 18 tyrosine residues.^[45]^ When a compound interacts with HSA, the fluorescence property of HSA decreases depending on the proximity of the compound to these amino acid residues.

#### Absorption and fluorescence spectral properties

The fluorescence spectra of HSA (1.0×10^−6^ M) and the absorption spectra of PYDP are shown in Figure 6. Upon excitation at 290 nm, the fluorescence spectra of HSA showed an emission maximum at 345 nm and the absorption spectra of PYDP showed maximum at 312 nm. We also measured the fluorescence spectra of HSA alone and in the presence of an increasing concentration of PYDP from 3.3×10^−5^ to 2.7×10^−4^ M. The results showed that the fluorescence intensity of HSA decreased significantly with increasing concentration of PYDP with a shift to high wavelengths, suggesting that there is an interaction between HSA and PYDP due to a non‐radiative energy transfer. The spectrum exhibited an isosbestic point at 382 nm.

#### Fluorescence quantum yields

One of the parameters used to quantify protein interactions in vivo and in vitro is quantum yield.^[46]^ As expected, the quantum yield of the donor should decrease when the acceptor is close to the donor for energy transfer to occur. We observed that the quantum yield of HSA substantially reduced with increasing concentrations of PYDP (Table 7), which is in the agreement with the result of spectral data.

**Table 7:** The fluorescence quantum yield of HSA with increasing concentration of PYDP in aqueous media.

[PYDP] / M	Φ_f_
0	0.445
3.33×10^−5^	0.228
6.67×10^−5^	0.116
1.00×10^−4^	0.064
1.33×10^−4^	0.040
1.67×10^−4^	0.024
2.00×10^−4^	0.020
2.33×10^−4^	0.020
2.67×10^−4^	0.008

#### Energy transfer parameters (J and R)

As shown in Figure 6, HSA has a broad fluorescence spectrum peak and overlaps very well with the absorption spectra of PYDP. Using these curves and quantum yields for water solutions, J and R were calculated. The distance is important for efficient energy transfer from donor to acceptor. Förster radius (R) was calculated as 29 Å for HSA‐PYDP pair, indicating that donor and acceptor are near, and FRET occurred with high probability. The value of overlap integral (J) for HSA ‐PYDP pair was found as 7.94×10^−13^ M^−1^ cm^−1^ nm^4^.

#### Fluorescence quenching mechanism (K_sv_, k_q_, K_a_, E, r(Å), n)

**Figure 7:**
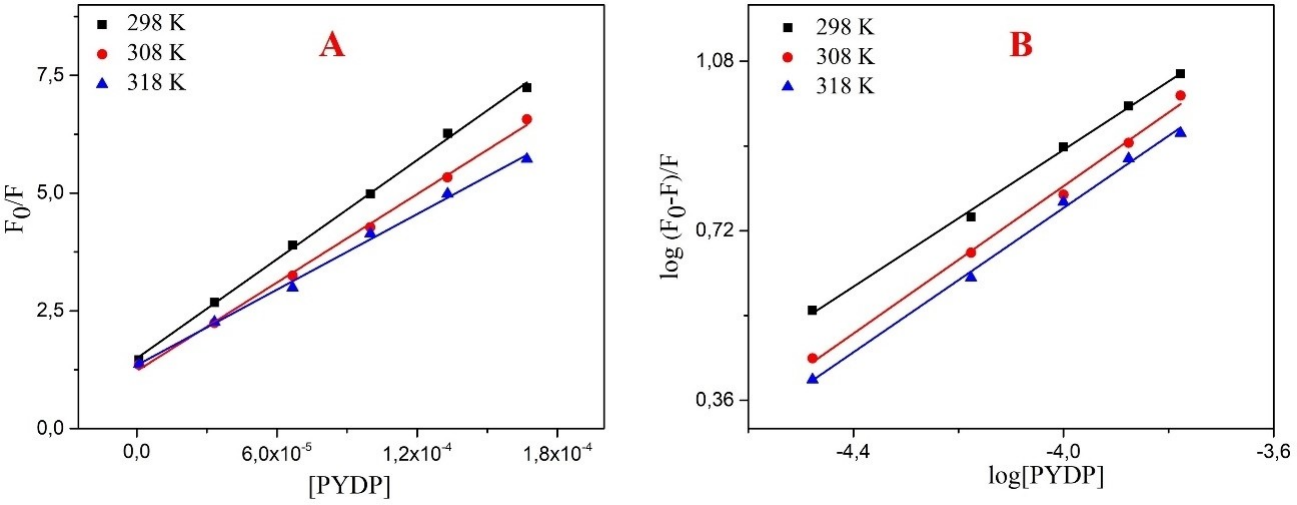
Stern‐Volmer plot and logarithmic Stern‐Volmer plot of HSA binding with PYDP in aqueous media.

To determine the quenching mechanism, we used the Stern–Volmer equation. The plots of F°−F/F vs. [Q] and log((F°−F))/F vs. log[Q] are given in Figures 7A and 7B, respectively. In the HSA‐PYDP pair, *K*_sv_ values were decreased with increasing temperature (from 25 °C to 45 °C). *K*_q_ is calculated from the Equation (6) and the fluorescence lifetime τ_0_ of HSA was reported approximately as 10^−8^ s.[^47^, ^48^] The k_q_ value is higher than quenching rate constant (1.0×10^10^ M^−1^ s^−1^), which indicates a diffusion‐controlled quenching mechanism.^[49]^ k_q_ values that appear larger than the diffusion‐controlled limit generally demonstrate some kind of binding interaction. The number of binding sites and the distance between the PYDP and HSA were similar at all temperatures (Table 8). The fact that the n values are close to 1 regardless of the temperature indicates that the interaction of HSA and PYDP is from a single region. It also means that the temperature increase does not damage the HSA‐PYDP interaction. FRET efficiency (E) depends on the distance of donor and acceptor, and the calculated E values were similar at different temperatures due to the comparable donor‐acceptor distance (Table 8).

**Table 8:** Stern‐Volmer association constants (*K*_sv_), quenching constants (k_q_), binding constants (*s*_a_), energy transfer efficiency (E), donor‐acceptor distance (r) and number of binding site (n), parameters of the interaction of PYDP with HSA at different temperatures (pH 7.40).

Temperature [°C]	*K*_sv_ [M^−1^]	k_q_ [M^−1^ s^−1^]	*K*_a_ [M^−1^]	E	r [Å]	n
25	3.50×10^4^	3.50×10^12^	6.31×10^3^	0.852	21.95	0.972
35	3.12×10^4^	3.12×10^12^	8.83×10^3^	0.823	22.74	0.978
45	2.67×10^4^	2.67×10^12^	6.82×10^3^	0.835	22.42	0.976

#### Fluorescence lifetime measurement studies

**Figure 8:**
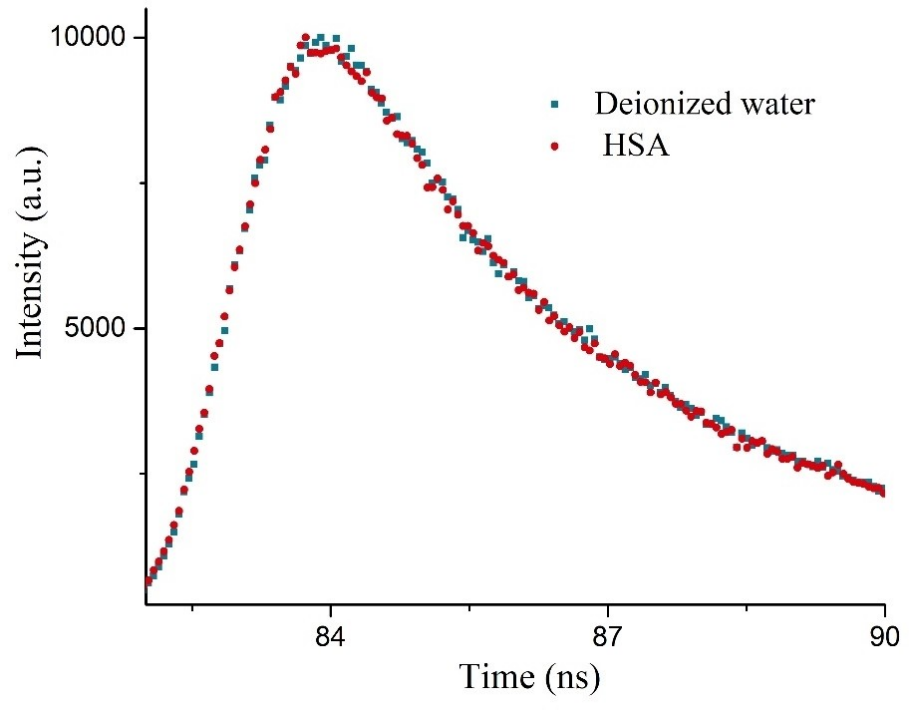
Fluorescence decay profile of HSA‐PYDP. [HSA]=1×10^−6^ M; λ_ex_=290 nm; λ_em_=305–570 nm and pH=7.4.

The fluorescence lifetime of HSA in the absence and in the presence of PYDP are shown in Figure 8. The fluorescence lifetime of HSA reduces in the presence of PYDP indicating the occurrence of energy transfer from the donor to the acceptor. The fluorescence lifetime profile of PYDP in aqueous media. The shorter (τ_1_) and the longer (τ_2_) fluorescence lifetime values of HSA were obtained as 1.618 ns and 4.300 ns, respectively. Since B_2_ (84.34 %) is greater than B_1_ (15.66 %), it is clear that the longer lifetimes are more apparent in the PYDP+HSA system (χ^2^: 1.403).

#### Thermodynamic parameters and the nature of the binding forces

The type and the magnitude of the binding forced are mainly determined by thermodynamic parameters.^[35]^ The Van't Hoff plot was used to determine the parameters including ΔH, ΔS and ΔG were estimated. ΔH^0^ and ΔS^0^ are found as −12.02 and 0.06, respectively. As the binding is enthalpically and entropically favorable (ΔH<0 and ΔS>0), it is more likely that the binding between HSA and PYDP occurs due to electrostatic/ionic interactions. We also found the value of ΔG^0^ to be −28.67, indicating that the binding occurs spontaneously.

### Cytotoxicity

**Figure 9:**
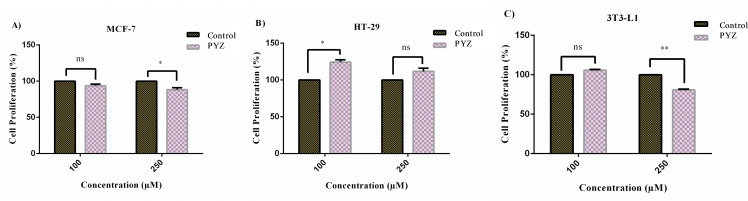
The cytotoxicity of the compound PYDP against human breast adenocarcinoma MCF‐7 (A), colorectal adenocarcinoma HT‐29 (B) and non‐carcinogenic mouse fibroblast 3T3‐L1 (C) cell lines. All the cell lines were exposed to the compound (PYDP) for 24 h. Each experiment was conducted in triplicate. ns(non‐significant); *p<0.05 (significant); **p<0.01 (highly significant).

Cytotoxicity studies are a mandatory step to introduce a newly synthesized chemical compound into the pharmaceutical industry and to evaluate it for various purposes in the biological systems, including the cell staining property of a probe.[^50^, ^51^, ^52^] As can be seen from Figure 9, PYDP at 100 μM (p>0.05) did not show a statistically significant change in the proliferation of MCF‐7 cells compared to cells in the control group. In contrast, a 12 % inhibition (*p<0.05) was observed for the HT‐29 cell line when the PYDP concentration was increased to 250 μM. The compound caused an increase in cell proliferation at 100 μM compared to the control group (*p<0.05), while at 250 μM (p>0.05) it caused not a statistically significant change on HT‐29. In the healthy cell line 3T3‐L1, PYDP caused no significant change in cell proliferation at 100 μM (p>0.05), but it caused statistically significant inhibition at 250 μM (**p<0.01). All these data show that PYDP has no cytotoxic effect on cancer and healthy cell lines at a concentration of 100 μM. On the other hand, at higher concentrations (250 μM), it still has no significant effect on cancer cells, while it has little cytotoxic effect on healthy cells.

### Confocal Microscope Examination

**Figure 10:**
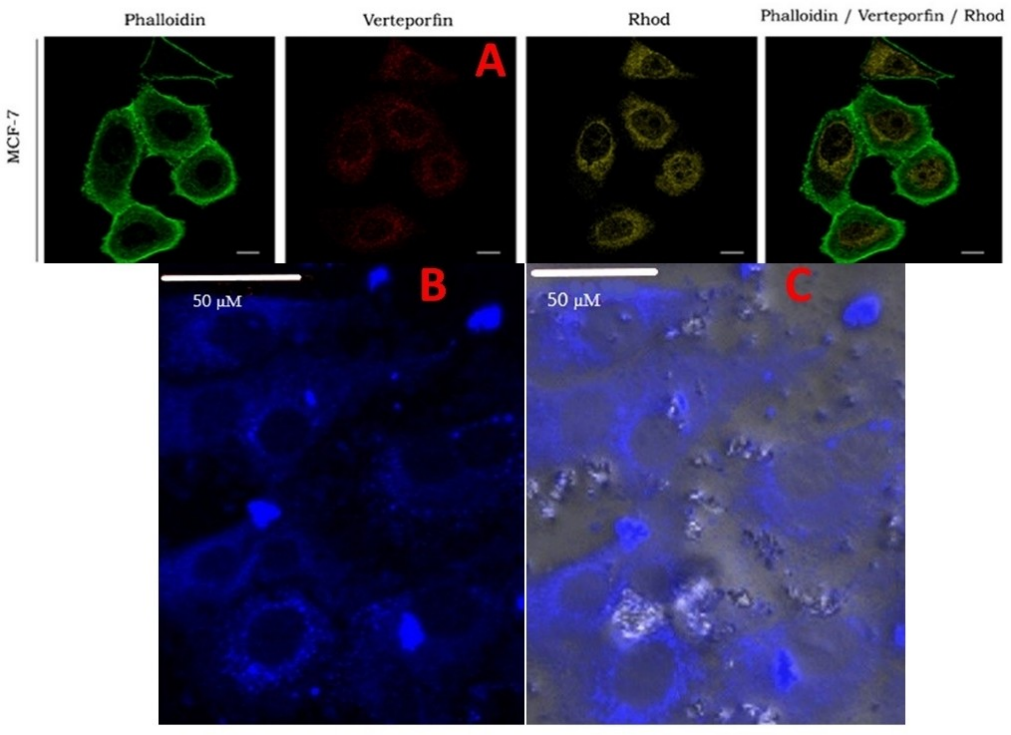
(A) Confocal microscopy image of MCF‐7 cell lines. Green fluorescence: Alexa Fluor® 488 Phalloidin; Yellow fluorescence: Rhodamine B‐conjugated Pluronics; Red fluorescence: Verteporfin. Scale bar=10 μm.^[53]^ (B) Confocal fluorescence microscopy image of MCF‐7 cell lines stained with PYDP, (C) merged of fluorescence and light microscopy image of MCF‐7 cell lines with PYDP.

From the application point of view, we investigated whether PYDP molecule could be used as a fluorescent dye to stain the fixed cell. For this purpose, MCF‐7 cells were fixed and stained with PYDP and observed by confocal microscopy (excitation at 350 nm and emission at 365–650 nm). The images obtained are shown in Figures 10B and 10 C. In Figure 10A, Green fluorescence of MCF‐7 cells from the literature: Alexa Fluor® 488 Phalloidin; Yellow fluorescence: Rhodamine B‐conjugated Pluronics; Red fluorescence: images stained with verteporfin have been given. Confocal microscopy images of MCF‐7 cell lines showed a cytoplasmatic distribution for both Verteporfin (red signal) and rhodaminated‐micelles (yellow signal). Besides, it is well known that preferential site of action of Verteporfin is the mitochondria. Here, pluronic micelles were used to photosensitizer Verteporfin.^[53]^ The images (Figures 10B and 10 C) show that PYDP penetrates the fixed cell membrane and exhibits fluorescence properties in both the cytoplasm and intracellular compartments. This suggest that the PYDP molecule could be used as a cytopainter. It is possible that the amine‐reactive group in PYDP binds covalently to cytoplasmic proteins, probably throughout the endoplasmic reticulum, allowing uniform cell imaging.^[54]^ It is clear from the images that PYDP does not stain the nucleus, which could mean that PYDP does not fluorescence in the presence of DNA.

## Conclusions

The document provides a detailed analysis of the interaction of PYDP with different dyes and HSA in aqueous media. PYDP was synthesized and characterized (by FTIR, ^1^H NMR, and ^13^C NMR spectroscopy), and investigated for its potential as a fluorescent probe using FRET based methods. Energy transfer parameters, quantum yields, quenching mechanisms as well as lifetime measurements have shown that PYDP acts as a donor for the dyes Safranin T, Acridine O, Pyronin Y, and Fluorescein dyes in SDS media and as an acceptor in HSA due to the overlapping spectra and the proximity between the molecules. The study involved the calculation of thermodynamic parameters. The results reveal that the interaction between PYDP and other fluorescent dyes was stable and spontaneously driven by negative ΔG^0^ values. Additionally, all donor‐acceptor pairs exhibited negative ΔH and positive ΔS values, indicating that all interactions were due to the electrostatic and ionic interactions. We also examined the cytotoxic effects of PYDP against MCF‐7 cell lines as well as the corresponding 3T3‐L1 cell lines using the XTT assay. This showed that PYDP has no cytotoxic effects on cancer and healthy cell lines at a concentration of 100 μM. However, at a higher concentration of 250 μM, it still has no significant effect on cancer cells, while it has a little cytotoxic effect on healthy cells. Finally, the cell staining property of PYDP was monitored using a confocal microscope. The results showed that PYDP penetrates the fixed cell membrane, exhibits fluorescence properties, and it selectively stained cytoplasm. This suggested that the PYDP molecule could be used as a cytopainter. Overall, the study provides valuable insights into the interaction of PYDP with different dyes and HSA in aqueous media. The findings of this study could have significant implications in the field of biophysical and physicochemical research and could pave the way for the development of novel fluorescent probes and cytopainters.

## Experimental Section

### General procedure for the synthesis of chalcone derivative 2‐(5‐(pyridin‐2‐yl)‐1H‐pyrazol‐3‐yl) phenol (PYDP)

**Scheme 1:**
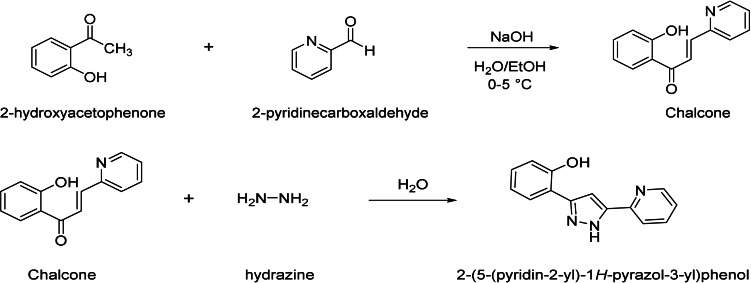
Synthesis procedure of 2‐(5‐(pyridin‐2‐yl)‐1H‐pyrazol‐3‐yl) phenol (PYDP).

PYDP was selected to investigate its potential as a fluorescent probe. The molecular structure and the synthesis of PYDP is described in Scheme 1. Chalcone derivative was synthesized by the reaction of 2‐hydroxyacetophenone with 2‐pyridinecarboxaldehyde in a yield of 67 %. Then, the reaction mixture was stirred in a microwave oven (800 watt) at 70 °C for 4 min using hydrazine hydrate to obtain the final product.^[28]^ Characterization of PYDP was carried out using FTIR, ^1^H NMR, and ^13^C NMR spectroscopy.

### Characterization of PYDP

Yield 90 %, dark brown oil (Column chromatography conditions: 50 hexane/40 : 10 hexane‐diethyl ether 30 : 20 H−E/20 : 30 H−E 40 : 10 H−E / 50 diethyl ether ; UV λ^CHCl3^_max_: 287 nm (ϵ, 7000). **FTIR** (KBr): NH:2921, C=N:1573, OH: 3395. ^**1**^ **H NMR** (200 MHz, CDCl_3_, *ppm*): 4.96 (t, *J*=24.0 Hz, *J*=12.0 Hz H‐3), 3.16 (dt, *J*=8.0 Hz, *J*=28.0 Hz H‐4a), 3.16 (dt, *J*=8.0 Hz, *J*=28.0 Hz, H‐4b), 6.16 (d, *J*=8.0 Hz, H‐3′), 6.83 (t, *J*=8.0 Hz, *J*=12.0 Hz, H‐4′), 7.31 (t, *J*=8.0 Hz, *J*=12.0 Hz, H‐5′), 7.83 (d, *J*=8.0 Hz, H‐ 6′), 8.57 (d, *J*=4 Hz, 1H, H‐3′′), 7.50 (d,, *J*=8.0 Hz, 1H, H‐4′′ ), 7.84 (t, *J*=8.0 Hz, *J*=16.0 Hz, 1H, H‐5′′), 7.61 (d, *J*=8.0 Hz, 6′′), 6.80 (s 1H ‐NH), 13.47 (s, 1H, ‐OH). ^**13**^ **C NMR** (50 MHz, CDCl_3_, *ppm*): 73.05 C3, 33.86 C4a, 33.86 C4b,151.52 C5, 125.22 C1′, 164.00 C2′, 118.56 C3′, 129.29 C4′, 120.26 C5′, 117.07 C6′, 149.04 C1′′ (C1), 149.04 C3′′ (C3), 126.93 C4′′ (C4), 137.40 C5′′ (C5), 122.94 C6′′ (C6).

### Chemicals and Solutions

All chemicals used in this study were obtained from Sigma‐Aldrich and they are all spectroscopic grade or HPLC grade. The cell lines being used for culture studies were purchased from ATCC (American Type Culture Collection).

Dye solutions (1.0×10^−3^ M) and PYDP solution (1.0×10^−4^ M) were prepared as stock solutions in a 2 : 1 ethanol‐chloroform mixture and kept in dark. In this study, the SDS concentration was prepared as 8.5 mM, close to the critical micelle concentration (8.1 mM).^[36]^ While preparing the phosphate buffer solution, a PBS tablet was dissolved in 200 mL of deionized water using an ultrasonic bath. HSA solutions were freshly prepared in phosphate buffer (1.0×10^−6^ M, pH 7.4). Solutions were prepared in deionized water, just before spectroscopic measurements.

### Experimental

For the experimental part, 1.0×10^−4^ M PYDP was pipetted and dried with argon, and dye solutions in increasing concentrations were added with automatic pipettes. Then the prepared SDS solution was added, and the total volume was adjusted to 4.0 mL. The samples were kept in an ultrasonic bath for 15 min before each measurement, so that the dye and PYDP were completely dissolved in the solution. The temperatures (25, 35 and 45 °C) were kept constant using a temperature‐controlled magnetic stirrer to survey the effect of temperature on UV and fluorescence data.

### Instrumentation

The UV‐ absorption spectra of dyes were monitored with T80+ UV/Vis spectrophotometer (PG Instrument). Fluorescence experiments were performed at 25°±0.1 °C using a Shimadzu RF‐5301 PC spectrofluorometer. Fluorescence lifetimes were determined Horiba‐Jobin‐Yvon SPEX Fluorolog 3–2iHR instrument equipped with time‐correlated single photon counting controller (TCSPC) at excitation wavelength of 310 nm and emission wavelength of 460–475 nm. Fluorescence measurements of each sample were recorded by repeating the study three times. Nuclear Magnetic Resonance (NMR) Spectroscopy analysis was performed with Bruker Ascend 400 MHz/54 mm ULH.

### In Vitro Cytotoxicity Assay

In vitro cytotoxic effects of PYDP on colorectal adenocarcinoma cell lines (HT‐29), breast cancer cell lines (MCF‐7) and mouse fibroblast (3T3‐L1) cell line were measured by the XTT method according to the manufacturer‘s instructions (Cell Proliferation XTT Kit, BI). The cells were seeded in 96‐well (Sarstedt, Numbrecht, Germany) at a density of 10,000 cells/well in a total volume of 100 μL. After 2 h of incubation (at 37 °C in a 95 % humidity and in an atmosphere containing 5 % CO_2_), the medium was removed from the adherent cells and the wells were washed with 50 μL PBS buffer. Then, 50 μL of fresh medium was added per well followed by 50 μL of the sample (PYDP) at two concentrations (100–250 μM). After 24 h of incubation, the medium in each well was removed. Then, 100 μL of basal medium and 50 μL XTT solution were added to each well. To the control wells, 100 μL of complete medium containing 0.2 % DMSO was added. The plate was placed in a CO_2_ incubator for 5–6 h. Finally, the absorbances at 450 nm (reference filter at 650 nm) were measured in an ELISA reader (Anthos‐HTII) and cell viability in the wells was calculated.

### Confocal Microscope images

MCF‐7 Cells were cultured in DMEM containing ultra glucose added with 10 % FBS, 1 % penicillin/streptomycin, and 200 mM L‐glutamine at 37 °C with 95 % humidity and 5 % CO_2_. When the cells reached 85–90 % stampede, the attached cells were trypsinized and deported from the culture flasks. They were carried onto the glass slides at an initial cell density of 1.0×10^5^ cells/well. After 24 h of incubation, the cells were fixed with 4 % paraformaldehyde for 30 min. Then, 100 μL of 100 μM PYDP was added to the cells and incubated in the dark for 15 min. These images of cells were obtained using a laser scanning confocal microscope (Zeiss LSM 710) at 20X magnification which the excitation wavelength was 360 nm and emission wavelength was 650 nm.

### Statistical analysis

Statistical analyses of the cytotoxicity assays were performed by one‐way ANOVA with unpaired t‐test using statistical program GraphPad Prism 6 (GraphPad, La Jolla, CA) Software 7.0). All results were expressed as means with their standard deviation (±SD). p<0.05 was considered as the minimum level of significance.^[55]^

## Conflict of interest

The authors declare that they have no known competing financial interests or personal relationships that could have appeared to influence the work reported in this paper.

1

## Data Availability

All data recorded and analyzed in this article are already included. Data will be made available based on reasonable request.
